# Conformational States of a Bacterial α_2_-Macroglobulin Resemble Those of Human Complement C3

**DOI:** 10.1371/journal.pone.0035384

**Published:** 2012-04-17

**Authors:** David Neves, Leandro F. Estrozi, Viviana Job, Frank Gabel, Guy Schoehn, Andréa Dessen

**Affiliations:** 1 Institut de Biologie Structurale (IBS), Université Grenoble I, Grenoble, France; 2 Centre National de la Recherche Scientifique (CNRS), Grenoble, France; 3 Commissariat à l'Energie Atomique (CEA), Grenoble, France; 4 Unit for Virus Host Cell Interactions UMI 3265 (UJF-EMBL-CNRS), Grenoble, France; Institut Pasteur Paris, France

## Abstract

α_2_ macroglobulins (α_2_Ms) are broad-spectrum protease inhibitors that play essential roles in the innate immune system of eukaryotic species. These large, multi-domain proteins are characterized by a broad-spectrum bait region and an internal thioester, which, upon cleavage, becomes covalently associated to the target protease, allowing its entrapment by a large conformational modification. Notably, α_2_Ms are part of a larger protein superfamily that includes proteins of the complement system, such as C3, a multi-domain macromolecule which is also characterized by an internal thioester-carrying domain and whose activation represents the pivotal step in the complement cascade. Recently, α_2_M/C3-like genes were identified in a large number of bacterial genomes, and the *Escherichia coli* α_2_M homolog (ECAM) was shown to be activated by proteases. In this work, we have structurally characterized ECAM by electron microscopy and small angle scattering (SAXS) techniques. ECAM is an elongated, flexible molecule with overall similarities to C3 in its inactive form; activation by methylamine, chymotrypsin, or elastase induces a conformational modification reminiscent of the one undergone by the transformation of C3 into its active form, C3b. In addition, the proposed C-terminus of ECAM displays high flexibility and different conformations, and could be the recognition site for partner macromolecules. This work sheds light on a potential bacterial defense mechanism that mimics structural rearrangements essential for activation of the complement cascade in eukaryotes, and represents a possible novel target for the development of antibacterials.

## Introduction

**Figure 1:**
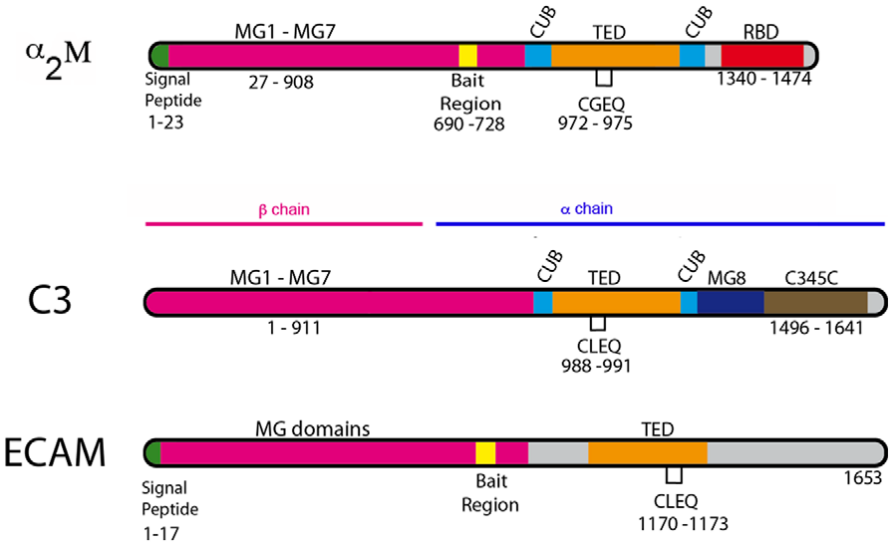
Schematic representations of human α-macroglobulin (α_2_M), C3 convertase (C3), and *E. coli* α-macroglobulin (ECAM). Domain assignments for α_2_M and C3 were based on their respective crystal structures [13], [17]. Assignments for ECAM were performed with the JPRED server, supported by the analysis performed by Doan and Gettins [27]. Note the similarity in domain predictions, including MG and TED domains. The CLEQ sequence, a signature of the thioester bond, is present in all proteins. For simplicity, only a limited number of the domains identified or predicted for the different molecules are depicted, and only one monomer of α_2_M is shown. The C-terminus of ECAM displays low sequence similarity to that of C3 (Fig. S6).

α_2_-macroglobulins (α_2_Ms) are large, multi-domain and broad-spectrum protease inhibitors present in eukaryotes that play key roles in host cell protection from parasitic or bacterial attack. These proteins are characterized by a highly reactive thioester bond as well as a bait region (Fig. 1) whose sequence is recognized by a large spectrum of proteases. The rearrangement of α_2_Ms upon cleavage of the bait region traps the attacking protease in a cage-like structure, thus rendering proteases secreted by infecting microorganisms ineffective, promoting efficient microbial clearance. α_2_Ms are thus essential elements of the innate immune system [1], [2].

The α_2_M bait region contains recognition sites for all four classes of proteases which, once physically entrapped, are impaired from reaching their substrates [2]. Human α_2_M, specifically, is a tetrameric 720 kDa molecule in which each 180 kDa subunit harbors an independent bait region whose cleavage induces the exposure and subsequent hydrolysis of a pre-concealed β-cysteinyl-glutamyl thioester bond. This generates an irreversible conformational modification causing one or two protease molecules to become entrapped within a cage-like structure [3], [4], [5], [6], [7], [8]. This modification also exposes the receptor-binding domain at the C-terminus of α_2_M, which is subsequently recognized by cells harboring the low density lipoprotein-related protein (LRP), allowing clearance of α_2_M-protease complexes [1], [2], [9]. Notably, the conformational change can also be induced *in vitro* through incubation of α_2_M with methylamine, which directly interacts with the thioester bond without altering the bait region [3], [4], [5], [7], [8], [10] and thus has been used extensively in the study of different forms of α_2_M molecules. Small angle scattering (SAXS) studies of human α_2_M revealed that the molecule becomes more compact when reacted with proteases and after incubation with methylamine [11], [12]. In addition, low-resolution electron microscopy data is available for α_2_Ms in both inactive and methylamine/protease-activated forms [3], [4], [5], [6], [7], [8], [10], and very recently, a medium resolution structure (4.3 Å) of the methylamine-activated human α_2_M also became available [13].

Notably, α_2_Ms are members of the same protein superfamily as components of the complement system, such as factor C3. In addition to displaying regions of considerable sequence similarity, these proteins harbor a number of homologous domains; most family members are characterized by a conserved CxEQ motif (Fig. 1), which forms the internal thioester bond that must become covalently associated to target molecules in order to ensure the protein's biological activity [1], [14], [15], [16]. The high-resolution crystal structure of the 187 kDa C3 molecule reveals that it is composed of two chains (α and β) divided into 13-domains, and that the highly reactive thioester is harbored within a protected region in the thioester-containing domain (TED) [17], [18]. The pivotal step in the complement cascade is the activation of C3 by proteolysis to yield C3b, in which the TED domain relocates to a site that is 75–100 Å away from its original position in C3. This exposes the thioester to solvent, allowing it to subsequently bind covalently to antigenic surfaces [19], [20], [21], [22], [23]; solvent-exposed Cys and Gln residues of the TED domain are also a feature of the human α_2_M [13]. It is thus evident that molecules of the α_2_M superfamily must undergo major conformational changes upon activation, and that these events are crucial for their biological activities.

Strikingly, α_2_M/C3-like molecules are not limited to metazoans. Sequence analyses of a number of bacterial genomes have recently identified α_2_M-like genes in several bacteria, most of which are pathogenic species and/or colonize higher eukaryotes [24], [25]. This allowed for the identification of two classes of bacterial α_2_Ms, with the most common one carrying the CxEQ motif and being encoded by a gene that is often located juxtaposed to the one coding for Penicillin-Binding Protein 1c (PBP1c). PBPs play key roles in the biosynthesis of peptidoglycan, a three-dimensional mesh that protects the bacterium from differences in osmotic pressure and gives it its shape [26]. This observation led to the suggestion that bacterial α_2_Ms could act in partnership with PBP1c during infection, the former protecting bacteria from proteases, the latter acting in cell wall repair upon potential disruption of the outer membrane and destruction of the peptidoglycan [24]. It is of note that disruption of the outer bacterial membrane could also occur in a non-infectious context, i.e., when members of the same bacterial community compete for nutrients. This suggests that α_2_Ms could be part of a bacterial defense mechanism. A second class of α_2_M, which in many species does not carry the CxEQ motif, was also identified amongst a large number of bacterial strains within an operon coding for four additional lipoproteins [24], but the function of this class of molecule is less clear.

*E. coli* carries both classes of α_2_Ms, and the mechanism of protease inhibition through a thioester-activation mechanism [2] was confirmed for the α_2_M from the PBP1c-related class. This protein was also shown to be modifiable by methylamine and proteases, much like eukaryotic α_2_M [27]. These findings reinforced the suggestion that bacteria, much like their eukaryotic counterparts, could employ α_2_M-like molecules to inhibit target proteases, thus facilitating the infection and colonization processes [24]. Notably, however, eukaryotic α_2_Ms have been reported to exist as dimers and tetramers [2], [8], whilst *E. coli* α_2_M is a monomer in solution [27]. This fact could facilitate the characterization of the bacterial form, as well as the detailed comprehension of its functionality. However, it is unlikely that the mechanism of protease targeting by bacterial α_2_Ms involves physical entrapment, due to its monomeric nature.

Here we report the structural characterization of α_2_M from *Escherichia coli* (henceforth called ECAM, in accordance with [27]) by small angle scattering (SAXS) and electron microscopy techniques in both native, methylamine-treated, and protease-activated forms. The overall shape of this monomeri α_2_M is highly reminiscent of that of C3, for which a high-resolution structure is available. Notably, SAXS experiments indicate that ECAM changes its conformation upon reaction with methylamine, chymotrypsin, or elastase. This modification is reminiscent of that observed for C3 upon activation to yield C3b [19], [20], [21], [22], [23] which exposes the thioester region. These results suggest that the mechanism of action of bacterial α-macroglobulins could involve recognition of proteases from the infected host, or secreted by competing bacterial species, through steps that are associated to a vast structural rearrangement.

## Results and Discussion

### Activated bacterial α_2_M highly resembles eukaryotic C3b

**Figure 2:**
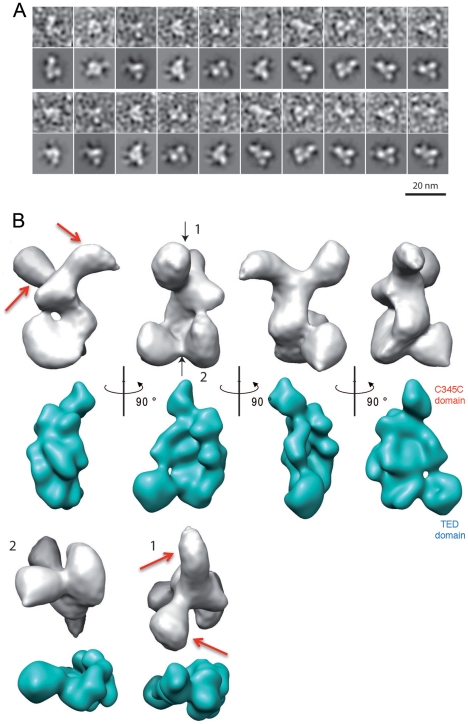
Electron microscopy reveals that ECAM is an elongated, flexible molecule. (A) Comparison between raw images, which were low-path filtered at 25 Å (lines 1, 3) and re-projections of the obtained 3D reconstructions (lines 2, 4). (B) Isosurface representations of the 3D reconstruction obtained for the activated form of ECAM (gray) and comparison with the 15 Å filtered structure of C3b (PDB 2I07, blue). For the isosurface representations, an averaged mass density of 0.84 Da/Å^3^ and a molecular weight value of 190 kDa were used. Black arrows represent top (1) and bottom (2) views, while the red arrows point to the regions of potentially greatest flexibility.

The α_2_M from *E. coli* (ECAM) is a 1653-residue molecule that carries a signal peptide, a lipoprotein box immediately following this sequence, and a multi-protease recognition (bait) region (Fig. 1). Sequence analyses using SMART (http://smart.embl-heidelberg.de) suggest the presence of multiple macroglobulin-like (MG) domains as well as a thioester-containing domain (TED), which are hallmarks of eukaryotic proteins of the α_2_M superfamily, including the well-studied C3 molecule. In order to obtain the first structural information of a bacterial α_2_M, we expressed ECAM in its soluble form (i.e., without the signal peptide or lipoprotein sequence) and activated it by treating with methylamine. This procedure yielded homogeneous samples of ECAM that were subsequently analyzed by negative staining electron microscopy employing sodium silico tungstate (pH 7.0, Fig. S1). In total, 51,700 individual particles were selected and aligned against the re-projections of a 30 Å-filtered model of C3 (PDB coordinates 2A73). This projection matching procedure yielded, after 50 cycles, a stable 3D model of ECAM with an estimated resolution between 15 and 20 Å (Fig. S1B). Notably, this 3D model showed clear similarities to the original images obtained by negative staining (Fig. 2A, lines 1 and 3; compare respectively with lines 2 and 4). In order to confirm that our 3D reconstruction was not model-biased, we performed image analysis by a reference-free classification (Figs. S2, S3). Comparison of the final ECAM activated 3D structure (Fig. 2B, gray) with that of C3b, filtered to 15 Å (Fig. 2B, blue), allows for the recognition of a number of key similarities, and one notable difference.

Methylamine-activated ECAM is an elongated molecule with overall dimensions of 140 Å×80 Å×80 Å, thus being reminiscent of the structure of C3b, whose dimensions are approximately 140 Å×80 Å×70 Å. Analysis of both the raw images and the re-projections of the 3D reconstructions (Fig. 2A and S1) suggest a molecule presenting 3 to 4 main regions of density, which could represent groups of domains, and a considerable level of flexibility. The latter point is also visible in the 3D models of ECAM shown in Fig. 2B, in which the top of the ECAM structure clearly shows two individual regions of electron density (indicated with red arrows). It is of note that only one of these protrusions is present in the filtered structure of C3b (blue, below); it is possible that this region, which corresponds to the C345C domain of C3b's α chain, is highly flexible in ECAM, and is positioned with two different conformations on the carbon grid, with both conformations being detected in the final structure. Attempts to individually characterize the two conformations were not successful, probably due to the relatively limited number of particles used in the 3D reconstruction (15,000). An alternative explanation to the existence of the two protrusions would be that one of them represents an additional domain present in ECAM but not in its eukaryotic counterparts; this seems unlikely, since sequence comparisons do not indicate the insertion of any large stretches of amino acids that would be required to generate a domain of this size.

Negative staining electron microscopy experiments of the native form were also performed, but a stable 3D model could not be obtained, probably due to a higher flexibility than for the activated form. Thus, in order to expand our study of the conformational changes undertaken by a bacterial α-macroglobulin during activation, we studied ECAM in native, methylamine-treated, and protease-activated forms by small angle X-ray scattering (SAXS) at physiological pH.

### ECAM changes conformation upon activation

**Figure 3:**
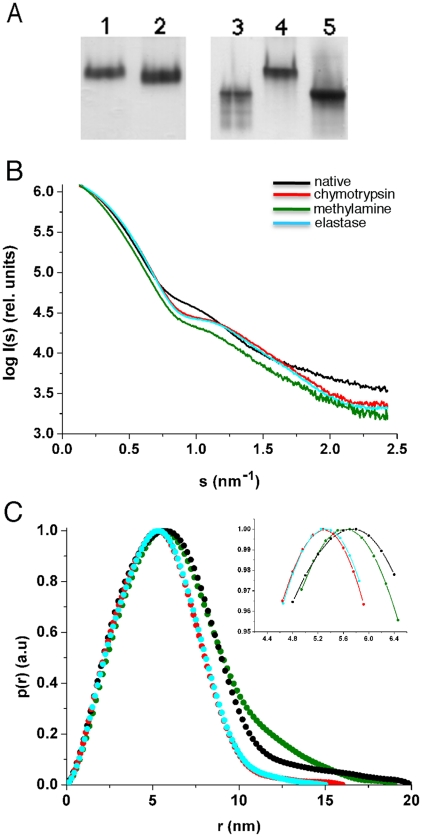
ECAM shows a change in gel mobility and overall structure upon activation. (A) Native PAGE showing the migration profile of ECAM. Lanes 1) native molecule; 2) methylamine-treated; 3) after reaction with chymotrypsin; 4) native molecule; 5) after reaction with porcine pancreatic elastase (B) Small-angle X-ray scattering (SAXS) results for native, methylamine-activated, elastase- and chymotrypsin-reacted ECAM. The radially averaged scattered X-ray intensity was plotted as a function of the momentum transfer *s*. Scattering patterns for ECAM in native form (black), after reaction with methylamine (green), elastase (blue) and chymotrypsin (red) were recorded in different concentrations (from 0.5 to 8 mg/mL) but only the curves relating to the highest concentration are shown. Inset, detail of differences in distinct side maxima. (C) Distance distributions *p*(r) of native, methylamine-reacted, elastase, and chymotrypsin of ECAM. All curves were normalized. Inset, detail of maxima of *p*(r) functions.

SAXS experiments were performed with four distinct samples: native ECAM, as well as ECAM reacted with methylamine, chymotrypsin, and elastase. All samples were purified by gel filtration chromatography. All activated forms of ECAM migrate faster than the native form in non-denaturing PAGE (Fig. 3A), suggesting that activation induces a conformational change and confirming the existence of electrophoretically ‘fast’ forms of bacterial α_2_Ms [27]. Notably, the transition from ‘slow’ to ‘fast’ forms by eukaryotic α-macroglobulins results in a considerable modification of the overall structure of the dimeric and tetrameric molecules, revealing that the interplay between bait region and thioester cleavage plays key roles in the induction of conformational changes [2].

Scattering patterns were recorded at different ECAM concentrations for all four samples and did not suggest any oligomerization or aggregation events, and are shown in Fig. 3B. The data are represented with the form log *I*(*s*) versus s (nm^−1^), where I is the measured intensity and *s* is the scattering angle. The intensity curve for native ECAM (black, Fig. 3B) shows a distinct side maximum that shifts to higher angles after the protein is reacted with methylamine and suggests that upon activation, ECAM undergoes a conformational change. A qualitatively similar change was also reported for the scattering curves of native and methylamine-treated human α_2_M [11], albeit on a different scale (ECAM is a monomer and human α_2_M a tetramer). In the case of the elastase or chymotrypsin-treated forms, the side maximum is shifted towards higher angles, indicating a compactation of the native structure, which is in agreement with the decrease of the maximum distance (*D*_max_) in the *p*(r) plots (Fig. 3C) from 19 to approximately 16 nm (Table 1). While the *D*_max_ of the native and methylamine-activated forms were similar, it follows from Fig. 3B that the main maximum in the *p*(r) curve displays a shift from 5.80 for native ECAM to 5.70 nm for the methylamine-treated form. In addition, there were significantly more differences in the range from 10 to 15 nm in the case of the methylamine-activated form with respect to the native form, which suggests a domain rearrangement in line with the increase of the *R*_g_ between both forms (Table 1). Interestingly, this change was more substantial after incubation with proteases, where the maxima were at 5.28 and 5.25 nm for the chymotrypsin and elastase complexes, respectively. Therefore, the modification in *D*_max_, the shape of the *p*(r) curve and a modified *R*_g_ all point to the fact that ECAM undergoes a conformational modification after reaction with methylamine, and this change is even more pronounced upon its reaction with proteases. Surprisingly, by employing fluorescence spectroscopy, Doan and Gettins recently concluded that ECAM does not undergo major structural modifications upon treatment with methylamine [27]. The reasons for this discrepancy are unclear, but the results presented here from both EM and SAXS studies clearly show that a conformational modification occurs upon activation.

**Table 1:** Structural parameters calculated from the models generated from SAXS measurements.

	*R*(_g_) (nm)	*D*_max_ (nm)
***SAXS***		
Native	4.67±0.01	20.0±2.4
Methylamine	5.14±0.03	19.0±1.9
Chymotrypsin	4.14±0.02	16.0±1.9
Elastase	4.14±0.01	15.0±2.0

The slow decline of the *p*(r) functions at large distances in all samples might suggest that parts of the structure can adopt a second, lowly populated conformation or structural flexibility; this effect is most pronounced for the native and methylamine activated forms (Fig. 3c). However, it should be noted that in the absence of high-resolution models of ECAM our SAXS data alone cannot provide a definitive answer. A more accurate Kratky plot representation revealed some slight structural flexibility that is most pronounced for the native form (Fig. S5). A potential interpretation could be that the C-terminal domain of ECAM can adopt several conformations in solution upon activation. This interpretation is supported by the EM observations/comparison with C3b (Fig. 2B) which show two potential positions for this domain (please see below).

### Conformational modifications of ECAM resemble those of eukaryotic C3 to C3b

**Figure 4:**
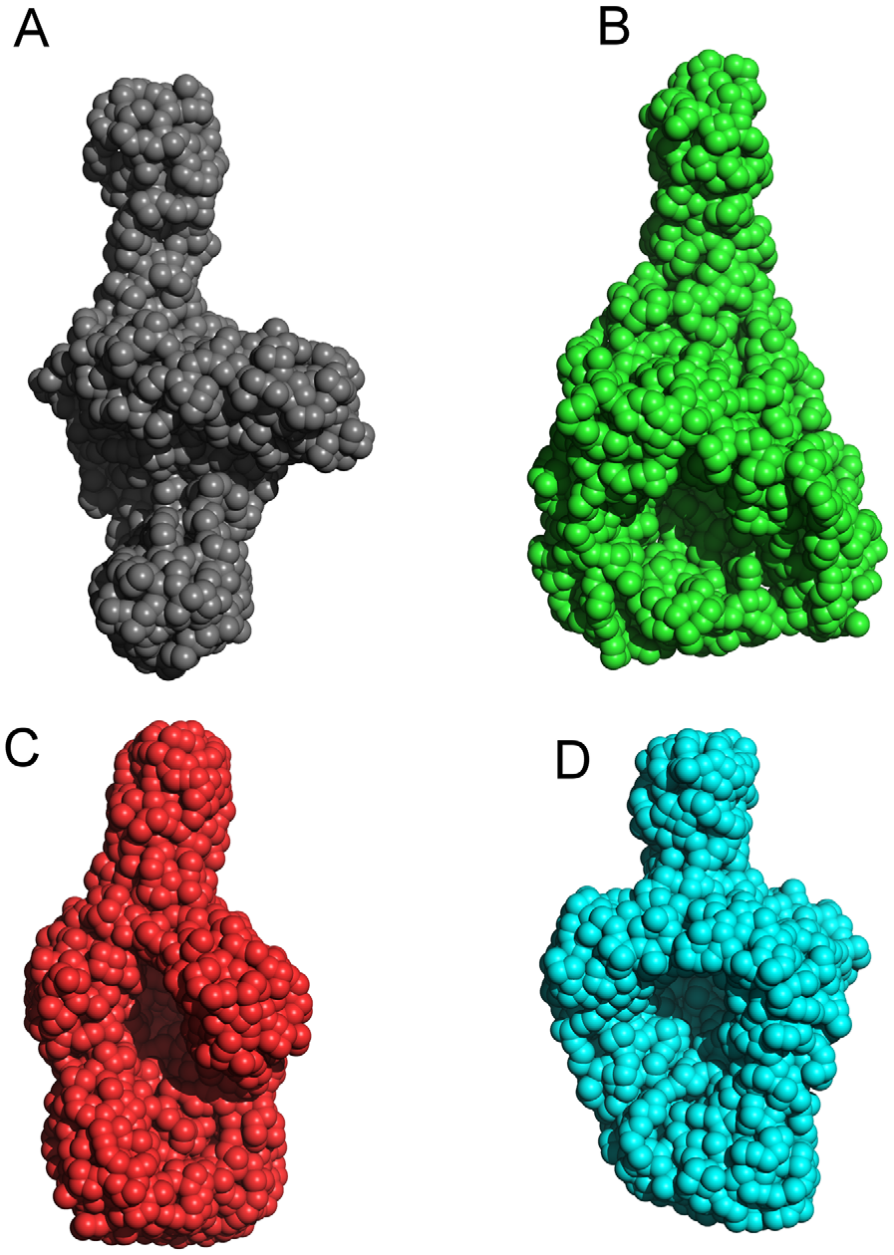
*Ab initio* models of ECAM generated by SAXS. Each model results from averaging 10 individual models calculated by the program GASBOR using: (A) native ECAM, (B) methylamine-treated, (C) chymotrypsin-treated, and (D) elastase-treated ECAM. Note the appearance of a central cavity in all of the treated forms of the molecule. GASBOR was used in “user" mode, following default options, except for the total number of residues, which corresponded to total ECAM (1653 residues). The envelopes are based on the *p*(r) funtions shown on Fig. 3, and the GNOM files generated were used as input for GASBOR. The models are drawn to scale.

**Figure 5:**
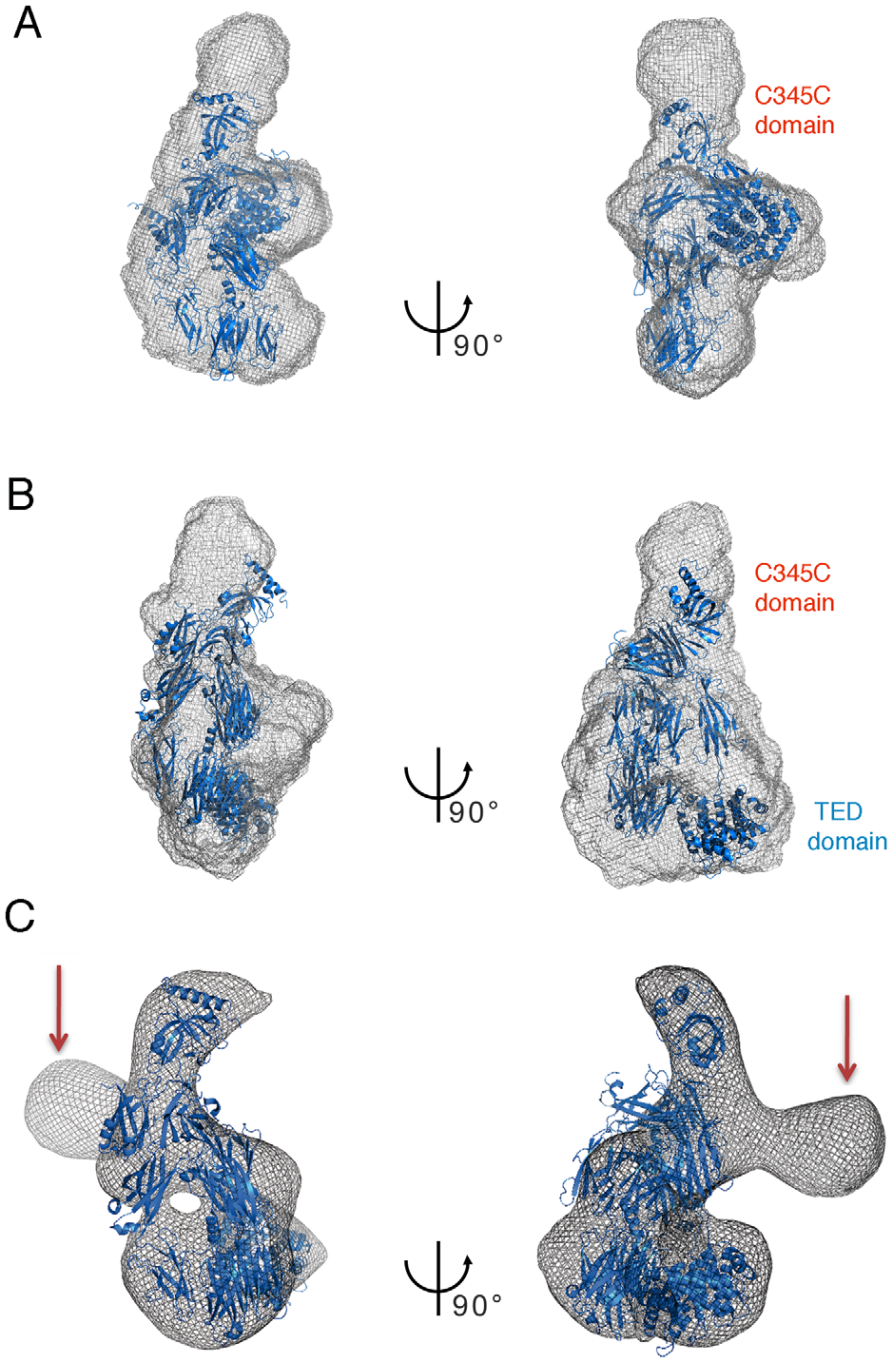
The structures of both native and activated forms of ECAM resemble those of C3 and C3b. Surface representations of ECAM in native (A) and methylamine-activated (B) states based on SAXS measurements. Structures of C3 (PDB 2A73) and C3b (PDB 2I07, in blue) were modeled manually into the ECAM *ab initio* SAXS models. Note that the difference in position of the C3/C3b TED (thioester-containing) domain can be well accounted for in the SAXS envelopes of both native (A) and methylamine-activated (B) forms of ECAM. (C) The X-ray structure of C3b (PDB 2I07) was manually fitted into the 3D EM envelope of the activated ECAM. The size of the macromolecule as well as the MG ring and the TED domain are in comparable positions. The unoccupied density, shown with red arrows, indicates the different potential position of the C-terminus domain and indicates high flexibility in this region. The lack of sequence similarity between the C-terminal domain of ECAM and C3b (Fig. S6) could also account for the differences observed.

Using the scattering data collected on ID14-3, we initially calculated models of native ECAM using GASBOR [28] with default options. After fifteen independent models were generated, they were averaged by DAMAVER [29]. Subsequently, a refined averaged model was calculated using GASBOR by employing a fixed core input file calculated by DAMSTART. The envelope of the methylamine-activated and protease-reacted forms of ECAM (Figs. 4B, C, D) indicate a clear conformational modification, generating a surface with a pear-like shape in all three cases. Notably, for all three forms, the conformational change generates what seems to be a cavity in the central part of the molecule. This feature is reminiscent of the ‘MG key ring’ reported in structures of C3b and other complement activation factors [19], [20], [21], [22], [23], [30]. Notably, in the C3 complement system, nucleophilic activation of the inactive thioester induces the TED and CUB domains to move away from the MG key ring, causing the thioester to become exposed; notably, in different structures of C3b, the final position of the TED domain is slightly modified, with respect to the angle that it makes with the rest of the structure [19], [20], [21], [22], [23], [30]. Thus, in order to explore the possibility that modification of the shape of ECAM from elongated into pear-like could correspond to a conformational change involving clear movement of the TED domain, we manually docked the structures of C3 and C3b onto the SAXS envelopes of native ECAM and methylamine-activated ECAM, respectively. The results are shown in Figs. 5A and 5B, where the envelopes are displayed as a gray mesh, and the structures of C3/C3b as blue ribbons. Results of similar structural comparisons using the program CRYSOL [31] are shown in Fig. S4. An initial observation that can be inferred from the abovementioned figures is that both C3 and C3b are similar to ECAM. Interestingly, in the native form of the molecule, one notices additional density for ECAM in a region that corresponds to the C-terminus of C3 (the C345C region). This extra density is also visible in the activated form of the molecule, albeit to a lesser extent.

The views shown in Fig. 5 strongly suggest that the modification in the surface of the activated form of ECAM could correspond to a change in the position of the TED domain, which, in C3b, is located between 75 and 100 A away from its position in C3 [19], [20], [21], [22], [23]. In order to gain further insight into this possibility, we manually fitted the structure of C3b (PDB 2I07, as above) onto the electron microscopy 3D model of methylamine-activated ECAM (Fig. 5C). This analysis reveals two important points. First, it corroborates the location the TED domain (as well as of the MG ring) in the activated form of the bacterial protein. In addition, this analysis suggests that the C-terminal region of C3b could be fitted into two different regions of density; only one was modeled, but the other potential conformation of the C-terminus of ECAM is indicated with red arrows. Thus, both SAXS and EM techniques point to the fact that the C-terminus of ECAM is potentially solvent-exposed and flexible. In eukaryotic α_2_Ms, the C-terminal, receptor-binding domain is exposed when the molecules are reacted with either methylamine or proteases, thus requiring a conformational modification for solvent accessibility [4], [7], [32]. This is also confirmed by the elegant docking of the structure of C3 and C3b onto electron microscopy maps of eukaryotic α_2_M, performed by Janssen and coworkers [20], as well as the recent 4.3 Å crystal structure of methylamine-activated human α_2_M [13]. This suggests that proteins of the α_2_M family share a number of overall structural similarities that include overall conformational modifications upon activation. It is of interest that inhibition of C3b by a Staphylococcal inhibitor protein occurs through the generation of an ‘open’ conformer of the former, which subsequently blocks formation of the C3 convertase [30], underlining the importance of complex conformational changes not only for C3 function but also for its targeting by pathogens.

The level of circulating α_2_M-protease complexes in humans is low, as a consequence of the recognition of the C-terminus of α_2_M by lipoprotein receptors and their subsequent internalization and degradation. Thus, the C-terminal region of eukaryotic α_2_M plays a key role in its recognition of partner macromolecules, leading to its eventual clearance [1], [2], [9]. The flexible C-terminal end of ECAM, described here, could also potentially serve as a binding region for partners. This could include PBP1c, whose gene co-occurs with that of α-macroglobulin in a number of bacterial species [24]. PBP1c is a periplasmic molecule that is anchored to the inner membrane through a single transmembrane region [26], [33]. The concerted action of PBP1c and ECAM could favor protection of cell integrity in the presence of foreign proteases [24], potentially through the involvement of a direct interaction between the PBP and the C-terminal region of the α-macroglobulin. This could reflect a novel bacterial defense mechanism that implicates the action of both protease inhibition and cell wall biosynthesis processes. On the other hand, pathogens have also been shown to encode proteins that mimic components of the complement system in order to manipulate the host inflammatory response [34], [35], [36]; thus, due to their similarity to C3/C3b, it is conceivable that bacterial α-macroglobulins could also play yet undefined roles in the disruption of the complement amplification pathway in situations where the outer cell wall is weakened. Either one of these potential mechanisms could represent unexplored targets for the development of novel antibacterials.

## Materials and Methods

### Materials

Porcine pancreatic elastase (PPE) (Fluka Biochemika) was dissolved in 0.2 M Tris-HCl pH 8.0. HisTrap HP, Superdex 200 10/300GL and Mono Q 5/50GL columns were purchased from GE Healthcare. Methylamine hydrochloride was obtained from ACROS Organics.

### Cloning, expression and purification of ECAM

The *yfhm* gene from *Escherichia coli* BL21 was amplified using conventional PCR methods and subsequently cloned into pet15b (Novagen), leading to a construct carrying a N-terminal polyhistidine tag and residues Asp19-Pro1653 of ECAM. The plasmid was transformed into BL21(DE3) and cells were grown in LB broth to an OD*_600 nm_* of 0.5–0.6 and induced for 3 h at 22°C with 0.5 mM isopropyl B-D-thiogalactoside. Unless otherwise stated buffer A (25 mM HEPES pH 7.5, 0.2 M NaCl) was used in all purification steps. After centrifugation of the cellular suspension at 5,000 g for 20 min at 4°C, the pellet was resuspended in buffer A complemented with anti-proteases leupeptin (0.5 µg/mL), aprotinin (0.7 µg/mL), PMSF (1.0 mM) and pesptatin (0.7 µg/mL). The lysate was obtained by sonication, centrifuged for removal of debris at 40,000 g for 40 min (4°C), and subsequently loaded onto a 5 mL HisTrap column in buffer A complemented with 50 mM imidazole. Protein was eluted with a single 250 mM imidazole step, and fractions were dialyzed overnight at 4°C against 25 mM HEPES pH 7.5, 10 mM NaCl. ECAM-containing fractions were subsequently loaded onto a Mono Q column equilibrated in the dialysis buffer and eluted with a linear gradient to 0.5 M NaCl. ECAM was further purified by gel filtration chromatography using a Superdex 200 column equilibrated in buffer A. The central fractions of the peak corresponding to ECAM were pooled and concentrated to 10 mg/mL.

### Preparation of activated forms of ECAM

Methylamine activation experiments were performed by incubating pure ECAM with 200 mM methylamine hydrochloride and 200 mM Tris-HCl pH 8.0 for 2 h at 25°C and subsequently subjecting the sample to gel filtration chromatography as described above. The central fractions of the peak were pooled and concentrated to 10 mg/mL.

The reactions with elastase and chymotrypsin were carried out using the same protocol. A 1∶1 molar ratio of protease∶ECAM was incubated at 37°C for 10 minutes, and the reaction was stopped with 1 mM PMSF, and subsequently injected on a gel filtration column; only the central fractions of the peak were used for further experiments.

### Small-angle X-ray Scattering

Measurements were recorded at the ID14-3 beamline of the European Synchrotron Radiation Facility (Grenoble, France). Prior to data collection a scattering curve of bovine serum albumin reference solution (5.2 mg/mL) was recorded. Experiments were performed at concentrations of 8.0 mg/mL for native ECAM, 6.4 mg/mL for the methylamine-activated form, 7.6 mg/mL for elastase-incubated ECAM and 6.1 mg/mL for the chymotrypsin-reacted ECAM. Between measurements, scattering from a buffer sample was recorded, and these data were subsequently subtracted from the respective sample curves. No radiation damage was observed during the ten 10 second exposure frames and all data were recorded at 25°C. Data were treated following default parameters of the PRIMUS software package [37]. The radius of gyration *R_g_* and the forward scattering value *I*(0) were estimated using the Guinier approximation [38]. Both parameters, as well the maximum particle dimension *D*_max_, were also calculated by the GNOM software [39]. *Ab initio* models of ECAM were generated using GASBOR [28]. A final average model was generated from 15 independent models using DAMAVER through their pairwise superposition [29].

**Electron microscopy** - Activated ECAM at a concentration of 0.05 mg/mL was first applied to the clean side of carbon on mica (carbon/mica interface) and negatively stained with 1% (w/v) sodium silico tungstate [40]. A grid was placed on top of the carbon film, and subsequently air-dried. Images were taken under low dose conditions with a Polara microscope (FEI Eindhoven, The Netherlands) operating at 300 kV and a nominal magnification of 59,000× with a Gatan CCD camera (USC 4000) (pixel size at the specimen level: 2 Å; defocus values ranging from 1.5 to 3 µm). A total of 51,700 particles were selected using a semi-automatic particle selection procedure with the EMAN boxer routine [41] and extracted into 100×100 pixel boxes. The images were CTF-corrected with CTFFIND3 [42] and Bsoft [43] and low-path-filtered to 25 and 10 Å. Due to the inherent flexibility of ECAM, only 15,000 particles were used to calculate the final 3D model, since the inclusion of a larger number of particles decreased the resolution.

### Image processing

A SPIDER [44]-based projection matching analysis using an initial 3D model calculated from the related C3 structure (PDB 2A73 filtered at 30 Å) was performed. A total number of 800 equally spaced re-projections (5 degrees sampling) and 50 cycles were used to obtain the final 3D reconstruction (the orientation parameters of the raw images and 3D reconstruction were stable). Each cycle consisted of model re-projections, alignment using the 25 Å low-path-filtered stack file, and 3D reconstructions using the 10 Å low-path-filtered stack file. Only the best 15,000 images (cross correlation criteria) were used during each cycle of projection matching. The resolution of the active form of the ECAM was found to be between 15 and 20 Å (0.3 and 0.5 Fourier-shell correlation criteria, respectively; Fig. S1B). A manual fit the C3b convertase (pdb 2I07) was performed using PYMOL.

### Reference-free image classification

A*b-initio* image classification was performed by using the IMAGIC software (ImageScience). After an initial centering step based on the average of all images, a multivariate statistical analysis (MSA) was performed by using a set of 69 eigen-images. The EM raw images were divided by similarity into 150 classes and the class averages were used to re-align/re-center the images; the above process was iterated 3 times, resulting in the classes presented in Fig. S2B. These classes were aligned against 196 equally distributed re-projections (every 10 degrees) of our reconstruction (Fig. S2A). The result is show in Fig. S3. Rows labeled 1 show the re-projections of our reconstruction whereas rows labeled 2 show the *ab initio* classes averages (same images as in panel S2b). For each re-projection, the corresponding *ab inito* calculated classes are very similar, confirming our 3D reconstruction.

## Supporting Information

Figure S1 A- Typical electron microscopy field of view obtained on SST negatively stained ECAM. B- Fourier shell correlation obtained by splitting the 15,000 images into halves in order to calculate two independent reconstructions (Yang et al., 2003).
(JPG)

Figure S2 Comparison between equally distributed re-projections (every 10 degrees; 196) of the EM 3D reconstruction (A) and the *ab-initio* obtained classes (150) (B).
(JPG)

Figure S3 Alignment of the 150 classes (Fig. S2B) against the 196 re-projections of our 3D reconstruction (Fig. S2A). The re-projections are shown in the rows labeled “1", and for each re-projection, the corresponding aligned classes are shown as a column (part 2).
(JPG)

Figure S4 Comparison of the SAXS data for both native and methylamine-activated ECAM with the crystal structures of C3 and C3b, respectively, using the program CRYSOL. Both fits are relatively good at low angles, indicating that the overall shapes (i.e., radii of gyration) are similar.
(TIF)

Figure S5 Kratky plot of SAXS experiments, which indicates that native ECAM is the form that displays the highest amount of flexibility.
(TIF)

Figure S6 Alignment of the C-terminal sequences of ECAM and C3, performed with the MUSCLE server. Identical residues are indicated with red boxes, and similar amino acids in gray. The CxEQ motif is indicated with diamonds. The two regions display 30% sequence similarity. Reference for FigureS6: Yang S, Yu X, Galkin VE, Egelman EH (2003). Issues of resolution and polymorphism in singleparticle reconstruction. J. Struct. Biol. 144, 162–171.
(PDF)
