# Supplementary figures and images for: Conformational States of a Bacterial α2-Macroglobulin Resemble Those of Human Complement C3
Source: PLoS One. 2012 Apr 17;7(4):e35384. doi: 10.1371/journal.pone.0035384 (PMC3328433; doi:10.1371/journal.pone.0035384)

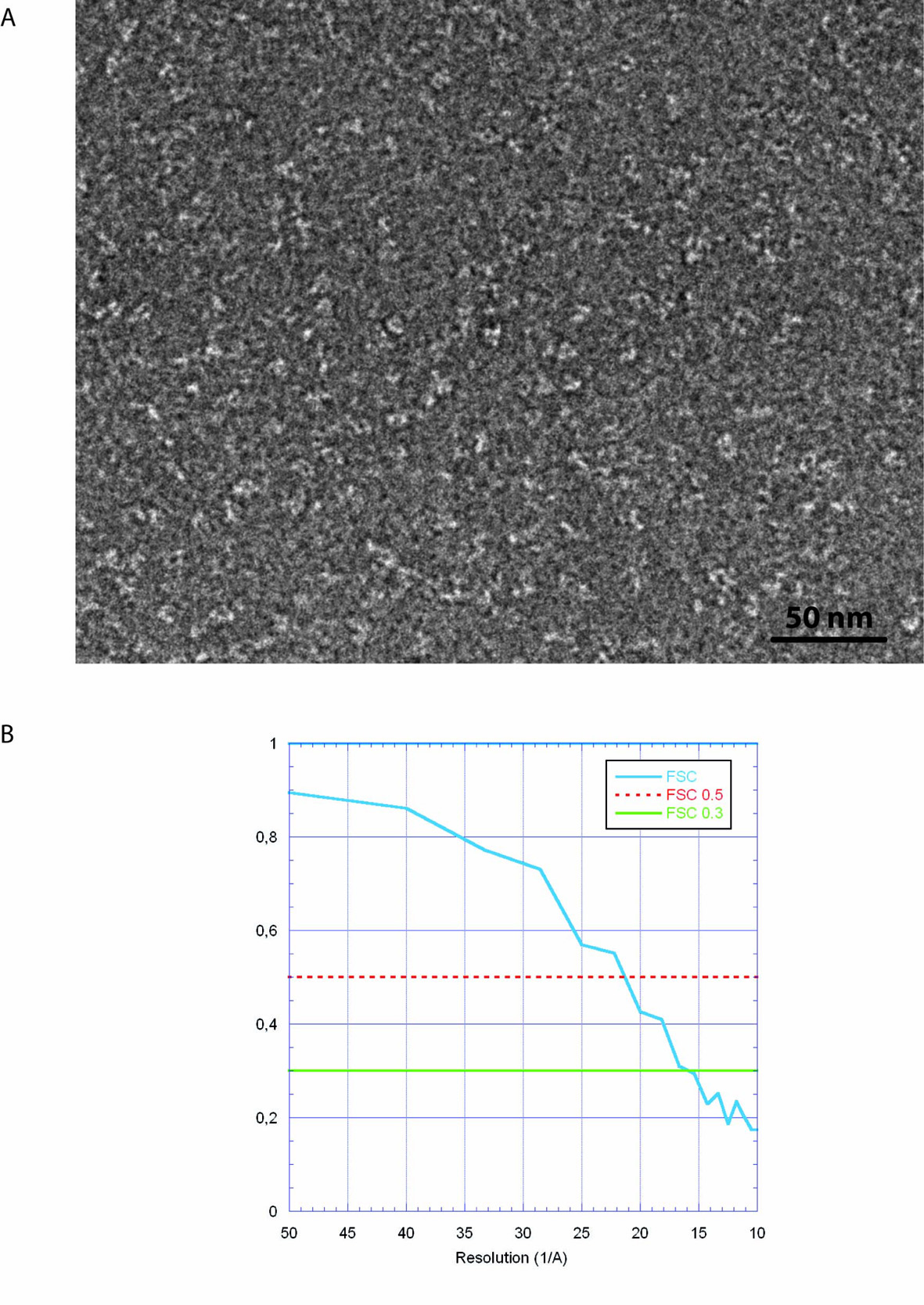

Supplement: Figure S1 — A- Typical electron microscopy field of view obtained on SST negatively stained ECAM. B- Fourier shell correlation obtained by splitting the 15,000 images into halves in order to calculate two independent reconstructions (Yang et al., 2003). (JPG) [file pone.0035384.s001.jpg]

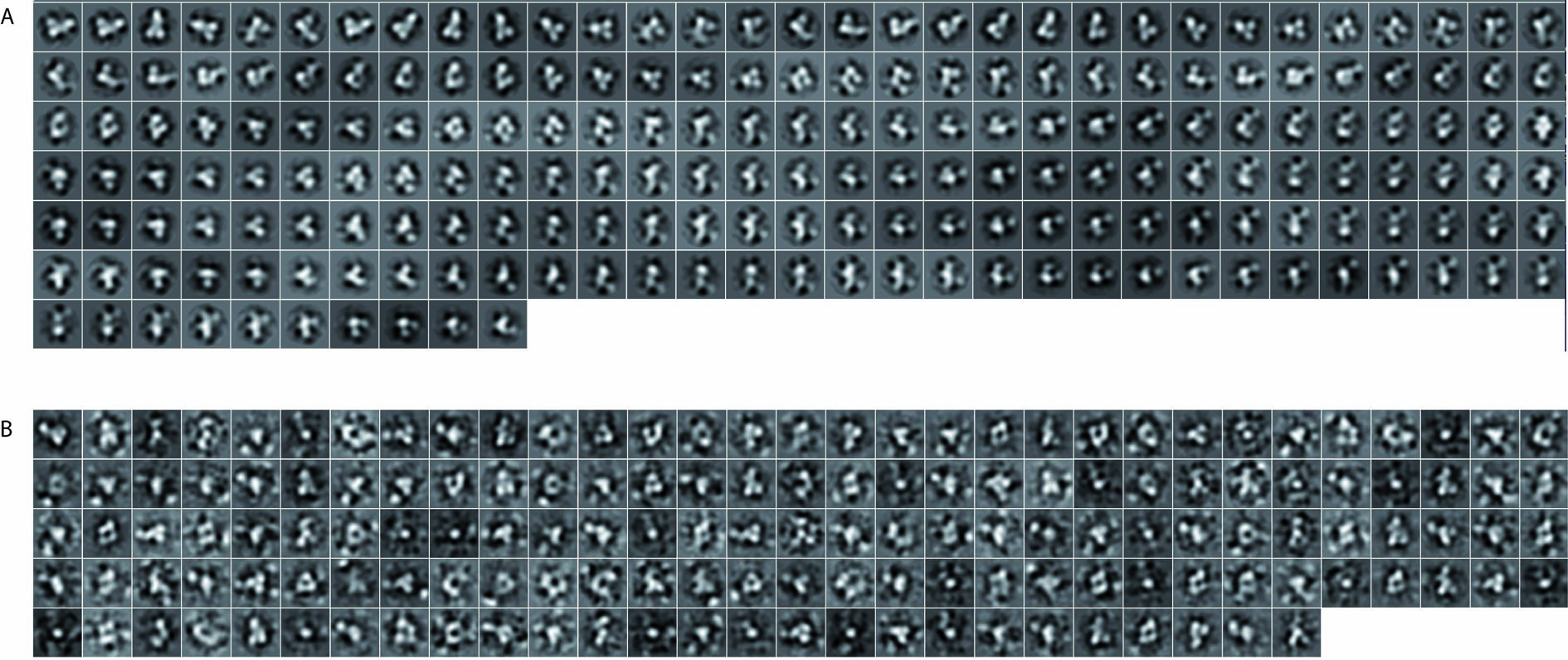

Supplement: Figure S2 — Comparison between equally distributed re-projections (every 10 degrees; 196) of the EM 3D reconstruction (A) and the ab-initio obtained classes (150) (B). (JPG) [file pone.0035384.s002.jpg]

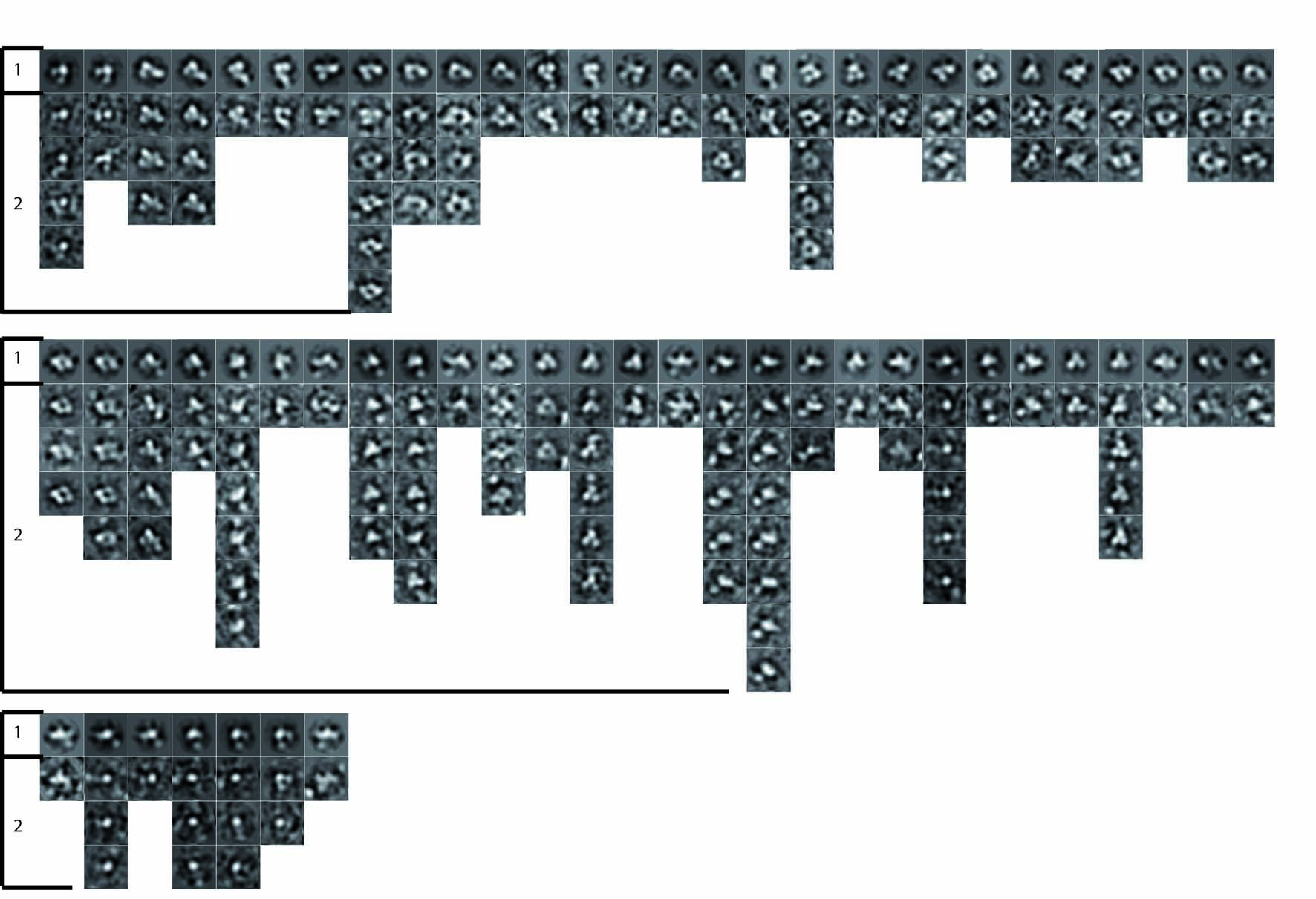

Supplement: Figure S3 — Alignment of the 150 classes (Fig. S2B) against the 196 re-projections of our 3D reconstruction (Fig. S2A). The re-projections are shown in the rows labeled “1", and for each re-projection, the corresponding aligned classes are shown as a column (part 2). (JPG) [file pone.0035384.s003.jpg]

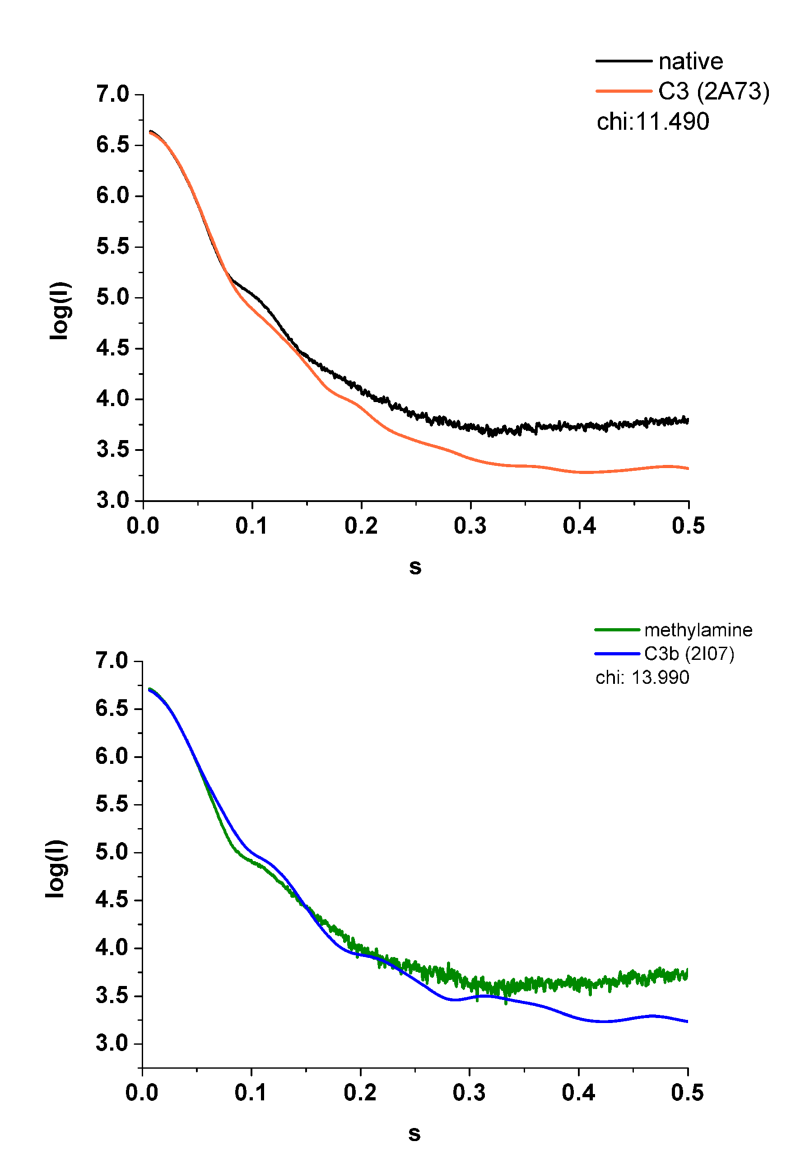

Supplement: Figure S4 — Comparison of the SAXS data for both native and methylamine-activated ECAM with the crystal structures of C3 and C3b, respectively, using the program CRYSOL. Both fits are relatively good at low angles, indicating that the overall shapes (i.e., radii of gyration) are similar. (TIF) [file pone.0035384.s004.tif]
