# Supplementary material for: Conformational States of a Bacterial α2-Macroglobulin Resemble Those of Human Complement C3
Source: PLoS One. 2012 Apr 17;7(4):e35384. doi: 10.1371/journal.pone.0035384 (PMC3328433; doi:10.1371/journal.pone.0035384)
Supplement: Figure S6 — Alignment of the C-terminal sequences of ECAM and C3, performed with the MUSCLE server. Identical residues are indicated with red boxes, and similar amino acids in gray. The CxEQ motif is indicated with diamonds. The two regions display 30% sequence similarity. Reference for FigureS6: Yang S, Yu X, Galkin VE, Egelman EH (2003). Issues of resolution and polymorphism in singleparticle reconstruction. J. Struct. Biol. 144, 162–171. (PDF) [file pone.0035384.s006.pdf]

```
                                                                 ♦ ♦♦
C3    VQKE-----DIPPADLSDQVPDTESETRILLQGTPVAQMTEDAVDAERLKHLIVTPSGCGEQNMIGMTPT 1021
ECAM  TALQPGETWAIPADGLQNFSPVT-LEGQLLLSGKPPLNI------ARYIKELKAYPYGCLEQTASGLFPS 1198

C3    VIAVHYLDETEQWEKFGL-----EKRQGALELIKKGYTQQLAFRQPSSAFAAFVKRAPST-WLTAYVVKV 1085
ECAM  LYT-----NAAQLQALGIKGDSDEKRRASVDI---GISRLLQMQRDNGGFALWDKNGDEEYWLTAYVMDF 1260

C3    FSLAVNL-IAIDSQVLCGAVKWLILEKQKPDGVFQEDAPVIHQEMIGGLRNNNEKDMALTAFVLISLQEA 1154
ECAM  LVRAGEQGYSVPTDAINRGNERLLR--------YLQDPGMMSIPYADNLKASKFAVQSYAALVLARQQKA 1322

C3    KDICEEQVNSLPGSITKAGDFLEANYMNLQRSYTVAIAGYALAQMGRLKGPLLNKFLTTAKDKNRWEDPG 1224
ECAM  ------PLGALREIWEHRAD---------------AASGLPLLQLGVA--------LKTMGDATRGEEA- 1362

C3    KQLYNVEATSYALLALLQLKDFDFVPPVVRWLNEQRYYGGGYGSTQATFMVFQALAQYQKDAPDHQELNL 1294
ECAM  -----------IALALKTPRN-----------SDERIWLGDYGSSLRDNALMLSLLEENKLLPDEQ---- 1406

C3    DVSLQLPSRSSKITHRIHWESASLLRSEETKENEGFTVTAEGKGQGTLSVVTMYHAKAKDQLTCNKFDLK 1364
ECAM  ---YTLLNTLSQQAFGERW-----LSTQESN-------------------ALFLAARTIQDLPGKWQAQ 1448

C3    VTIKPAPETEKRPQDA--KNTMILEICTRYRGDQDATMSILDISMMTGFAP-DTDDLKQLANGVDRYISK 1431
ECAM  TSFSAEQLTGEKAQNSNLNSDQLVTLQVSNSGDQPLWLR-MDASGYPQSAPLPANNVLQIERHILGTDGK 1517

C3    YELDKAFSDRNTLIIYLD-KVSHSEDDCLAFKVHQYFNVELIQPGAVKVYAYYNLEESCTRFYHPEKEDG 1500
ECAM  SKSLDSLRSGDLVLVWLQVKASNSVPDALV--------VDLLPAGL------------------ELENQ 1560

C3    KLNKLCRDELCRCAEENCFIQKSDDKV-TLEERLDKACEPGVDYVYKTRLVKVQLSNDFDEYIMA-IEQT 1568
ECAM  NL-----------ANGSASLEQSGGEVQNLLNQMQQASIKHIEF-RDDRFVAAVAVDEYQPVTLVYLARA 1618

C3    IKSGSDEVQVGQQRTFISPIKCREALKLEEKKHYLMWGLSSDFWGEKPNLSYIIGKDTWVEHWPEEDECQ 1638
ECAM  VTPG----------TYQVPQPMVESM-------------------------------YVPQW------- 1639

C3    DEENQKQCQDLGAFTESMVVFGCPN 1663
ECAM  --------RATGAAEDLLIVRP--- 1653
```
